# Obsessive-compulsive disorder onset and COVID-19 pandemic: Is there a relation between both?

**DOI:** 10.1192/j.eurpsy.2021.1966

**Published:** 2021-08-13

**Authors:** J. Aldeias, F. Polido, M.D.C. Cruz

**Affiliations:** Psychiatry, Centro Hospitalar Universitário do Algarve, Portimão, Portugal

**Keywords:** Obsessive-compulsive Disorder, COVID-19 pandemic

## Abstract

**Introduction:**

Obsessive-Compulsive Disorder (OCD) is a disorder in which a person has uncontrollable, reoccurring thoughts (obsessions) and/or behaviors (compulsions). Since the COVID-19 pandemic started, a lot of people developed fears of contamination or being infected.

**Objectives:**

To describe a clinical case and discuss the diagnosis of Obsessive-Compulsive Disorder in context of COVID-19 pandemic.

**Methods:**

The data was achieved through patient’s and his family. For the literature review we searched the terms: “OCD” and “COVID-19 Pandemic”.

**Results:**

45 years-old, male, single. He has a generalised anxiety disorder since 2010. At July 2020, the patient asked for help due to worsening of symptoms. Before the appointment, he was waiting outside because he didn’t feel comfortable in the waiting room. When he touch anything unintentionally, he wash his hands immediately. Since the COVID-19 pandemic began, he stopped working because he was too afraid of being infected. He started to think a lot about SARS-COV2 contamination, avoiding all public places, depending on his mother and friends to do basic daily tasks. His thoughts led to cleaning and hand-washing rituals, spending a lot of time. Those symptoms are egodystonic, have a huge impact on global functioning and are not explained by normative fear or protection measures. During 10 years of psychiatric follow-up he never showed obsessive-compulsive symptoms.

**Conclusions:**

This case is an example of OCD onset during the COVID-19 pandemic in a patient with an anxiety disorder (without previous obsessive-compulsive symptoms). He has improved after paroxetine 60mg and risperidone 1mg daily, as well as cognitive behaviour therapy weekly.

**Disclosure:**

No significant relationships.

